# EEG-based stroke severity classification using higher-order topological features and graph convolutional networks

**DOI:** 10.3389/fnins.2026.1791960

**Published:** 2026-04-17

**Authors:** Lu Zhang, Hanwen Zhang, Xiaomeng Fan, Qiang Li

**Affiliations:** 1 Institute of Science and Technology for Brain-Inspired Intelligence, Fudan University, Shanghai, China; 2 Department of neurology, Gongli Hospital of Shanghai Pudong New Area, Shanghai, China

**Keywords:** brain network, cycle ratio, electroencephalography, graph convolutional network, persistent homology, stroke severity, topological data analysis

## Abstract

**Introduction:**

Electroencephalography (EEG)-based stroke analysis has mainly relied on conventional signal and network descriptors, while higher-order brain network structures remain insufficiently characterized.

**Methods:**

We used persistent homology to extract cycle-based topological features from EEG functional networks, capturing higher-order organization with reduced sensitivity to threshold selection. These features were integrated with conventional EEG representations and embedded into a graph convolutional network for stroke severity classification.

**Results:**

The proposed framework achieved 86% accuracy in discriminating mild from moderate stroke. Cycle ratio analysis further revealed that the prefrontal cortex exhibited the most prominent higher-order structures, indicating its prominent involvement in post-stroke brain network organization.

**Discussion:**

Our results suggest that higher-order topological features can enhance EEG-based stroke severity classification and offer additional insight into post-stroke brain network alterations.

## Introduction

Stroke is a leading cause of death and disability worldwide (Tao et al., 2020), posing a significant burden on public health (Feigin et al., 2014; Rochmah et al., 2021). Early and accurate diagnosis is crucial for guiding effective treatment (Saposnik et al., 2011; McArthur et al., 2011; Powers et al., 2019). However, the subjective nature of clinical evaluations, which often depends on the expertise of medical professionals, can introduce variability in diagnoses. This variability underscores the need for objective, scientifically grounded methods to assess stroke severity, enabling precision medicine in stroke management.

EEG is a valuable tool for understanding the neural mechanisms of stroke and assessing its impact on brain function. Unlike other diagnostic methods, such as computed tomography or magnetic resonance imaging, EEG is non-invasive, cost-effective, and provides real-time, high-temporal resolution data. These advantages make EEG particularly useful for monitoring brain activity, detecting neural oscillation abnormalities, and evaluating functional impairments that may not be visible on structural imaging (Monge-Pereira et al., 2017; Babiloni and Astolfi, 2014).

The existing research on EEG-based stroke feature extraction mainly focuses on time-domain, frequency-domain, and traditional brain network features. Mulyanto et al. classified stroke severity based on statistical differences in time-domain features (Mulyanto et al., 2019). (Wilkinson et al. 2020) used frequency-domain features and decision tree classifier, achieving a prediction accuracy of 76% for stroke severity. (Nurfirdausi et al. 2022) employed frequency-domain features and three classifiers: k-nearest neighbors, decision trees, and naive Bayes, to categorize stroke severity into four classes, achieving a maximum accuracy of 83%. (Vecchio et al., 2019) explored the correlation between the small-world characteristics of the brain network and stroke severity using statistical methods.

Currently, little research has focused on higher-order structures, such as cycles and cavities, in the brain networks of stroke patients. Higher-order structures represent the global features of the brain and are less sensitive to threshold variations in constructing brain networks (Lee et al., 2011). El-Yaagoubi et al. proposed a method combining causal inference and hodge decomposition, known as causality-based topological ranking. This approach accurately and consistently identifies hierarchical structures within multivariate time series data and has been successfully applied to analyze changes in interactions among brain regions during epileptic seizures using EEG data (El-Yaagoubi et al., 2024). (Chung et al. 2024), in their study on maltreated children, utilized persistent homology along with the wasserstein distance to reveal that maltreatment may increase the homogeneity of white matter structures, leading to higher correlations in structural covariance. They also employed persistent homology to compare non-amnestic and amnestic Alzheimer's disease patients, uncovering more extensive white matter degeneration and lower structural connectivity in the former group (Phillips et al., 2024). Furthermore, (Jafadideh and Asl 2022) used persistent homology to show that autism spectrum disorder patients have more segregated and isolated networks in specific dynamic functional connectivity states, reflecting disruptions in network integration. These studies highlight the unique insights provided by persistent homology in understanding the topological properties of brain networks and their alterations in various neurological conditions.

In conclusion, existing EEG-based stroke studies typically construct brain networks by applying a hard threshold to correlation matrices, a process that is inherently subjective and sensitive to threshold selection. Moreover, such pairwise connectivity measures fail to capture higher-order network structures (e.g., cycles) that reflect multi-node interactions and global information integration. To date, no study has investigated the role of these higher-order topological features in EEG-based stroke severity classification. To bridge this gap, we introduce persistent homology—a technique that extracts cycle-based features independent of threshold choice—and integrate them with conventional EEG descriptors. This work provides the first evidence that incorporating higher-order topological information can enhance stroke severity classification and reveal clinically meaningful network reorganization, such as the prominent involvement of prefrontal regions in cycle structures.

The pipeline of our stroke severity classification includes four main steps. First, EEG signals are preprocessed, segmented, and transformed into functional connectivity graphs using Pearson correlation coefficient and phase-locking value. Second, for each EEG channel, we extract conventional time-domain, frequency-domain, and local network features together with higher-order topological descriptors derived from persistent homology. Third, informative features are selected and normalized, and class imbalance is addressed using SMOTE. Finally, the resulting graph-structured representations are fed into a lightweight graph convolutional network (EEGGCN1) for mild-versus-moderate stroke classification. This framework is further evaluated through cross-validation, external validation, and sensitivity analyses, aiming to determine whether higher-order topological features improve EEG-based severity discrimination and provide additional insight into stroke-related functional network alterations.

## Methods

We adopted a Graph Convolutional Network because it is particularly suitable for the proposed topological features, as it is inherently designed to process graph-structured data. The higher-order features derived from persistent homology encode the intrinsic topology of brain networks, and the GCN effectively aggregates this spatial and relational information through its message-passing mechanism, enabling the model to capture both local and global patterns of brain connectivity.

### Experimental data

**Figure 1:**
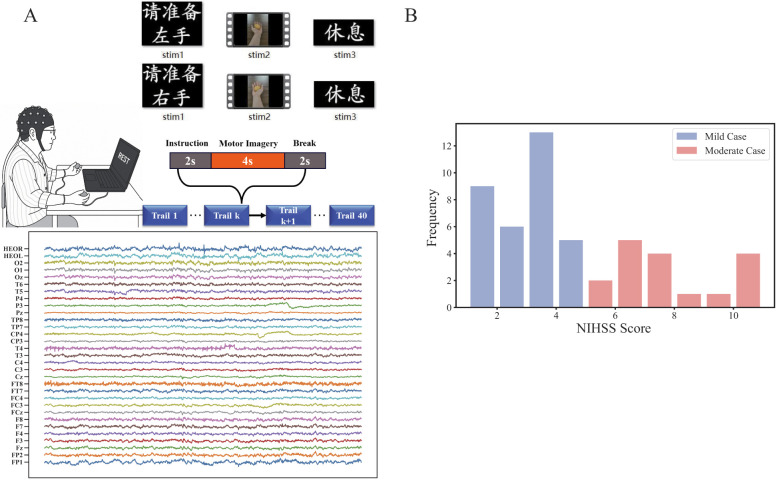
Motor imagery of left and right hand and data acquisition. **(A)** EEG experiment and signals. **(B)** NIHSS for mild and moderate patients. Data were obtained from (Liu et al. 2024) and used to generate the figure in this work.

We utilized the publicly available EEG motor imagery (MI) dataset for brain-computer interface in acute stroke patients (Liu et al., 2024). As shown in Figure 1A, the MI experiment consisted of 40 trials, each lasting 8 seconds and divided into three phases: instruction, MI, and break. 29 electroencephalogram (EEG) channels and 2 electrooculogram (EOG) signals were recorded during the MI experiment.

In our study, patients were categorized into two groups—mild (33 patients) and moderate (17 patients)—based on the National Institutes of Health Stroke Scale (NIHSS). For this study, the scale was applied in its original form without modification. Patients with NIHSS scores ≤ 4 were classified into the mild group, whereas those with 5 ≤ NIHSS scores ≤ 15 were classified into the moderate group (Pournajaf et al., 2023; Olive-Gadea et al., 2023; Yakhkind et al., 2016; Lee et al., 2022). The demographic and clinical information of the patients did not show statistically significant differences in age, sex, duration, affected hand, first onset, stroke location or lesion region were found between the mild and moderate groups (Table 1). The distribution of NIHSS scores is shown in Figure 1B, which clearly separates the mild and moderate groups.

**Table 1:** Baseline characteristics of mild and moderate ischemic stroke patients.

Variable	Mild (*n* = 33)	Moderate (*n* = 17)	Statistic	*p*-value
Continuous variables				
Age (years), mean ± SD	57.58 ± 10.37	55.00 ± 11.07	*t* = 0.7959	0.4322
Duration (days), mean ± SD	5.030 ± 5.324	7.235 ± 9.277	*U* = 265.5	0.7640
NIHSS, mean ± SD	2.424 ± 1.062	7.529 ± 2.095	*U* = 0.000	< 0.001
Categorical variables				
Sex (female/male)	8/25	3/14	–	0.7278
Affect hand (left/right)	19/14	9/8	–	0.9904
First onset (no/yes)	9/24	5/12	–	1.000
Stroke location (bilateral/left/right)	5/12/16	0/8/9	–	0.2298
Lesion region (Basal ganglia & Subcortical
/Brainstem/Cortex/Other)	15/12/3/3	8/7/2/0	–	0.6369

Statistical notes: For continuous variables, the Shapiro—Wilk test was performed within each group to assess normality. If both groups satisfied the normality assumption (*p* > 0.05), Welch's *t*-test was used to compare means. If normality was violated in either group (*p* ≤ 0.05), the Mann—Whitney *U* test was applied to compare distributions. For categorical variables, the Chi-square test was initially used to assess differences in distribution. If any expected cell count was < 5, Fisher's exact test was applied for 2 × 2 contingency tables; for contingency tables larger than 2 × 2, the Chi-square test was retained.

The data used in this study included only the first 20 trials for each patient to minimize artifacts resulting from prolonged sitting postures. Independent component analysis was applied to the preprocessed data to further eliminate oculographic and myoelectric artifacts. The EEG signals were then divided into five frequency bands: delta (1—3 Hz), theta (4–7 Hz), alpha (8–12 Hz), beta (13–30 Hz), and gamma (31–40 Hz). Finally, 29 electrode channels were selected to represent brain nodes.

### Graph convolutional network

**Figure 2:**
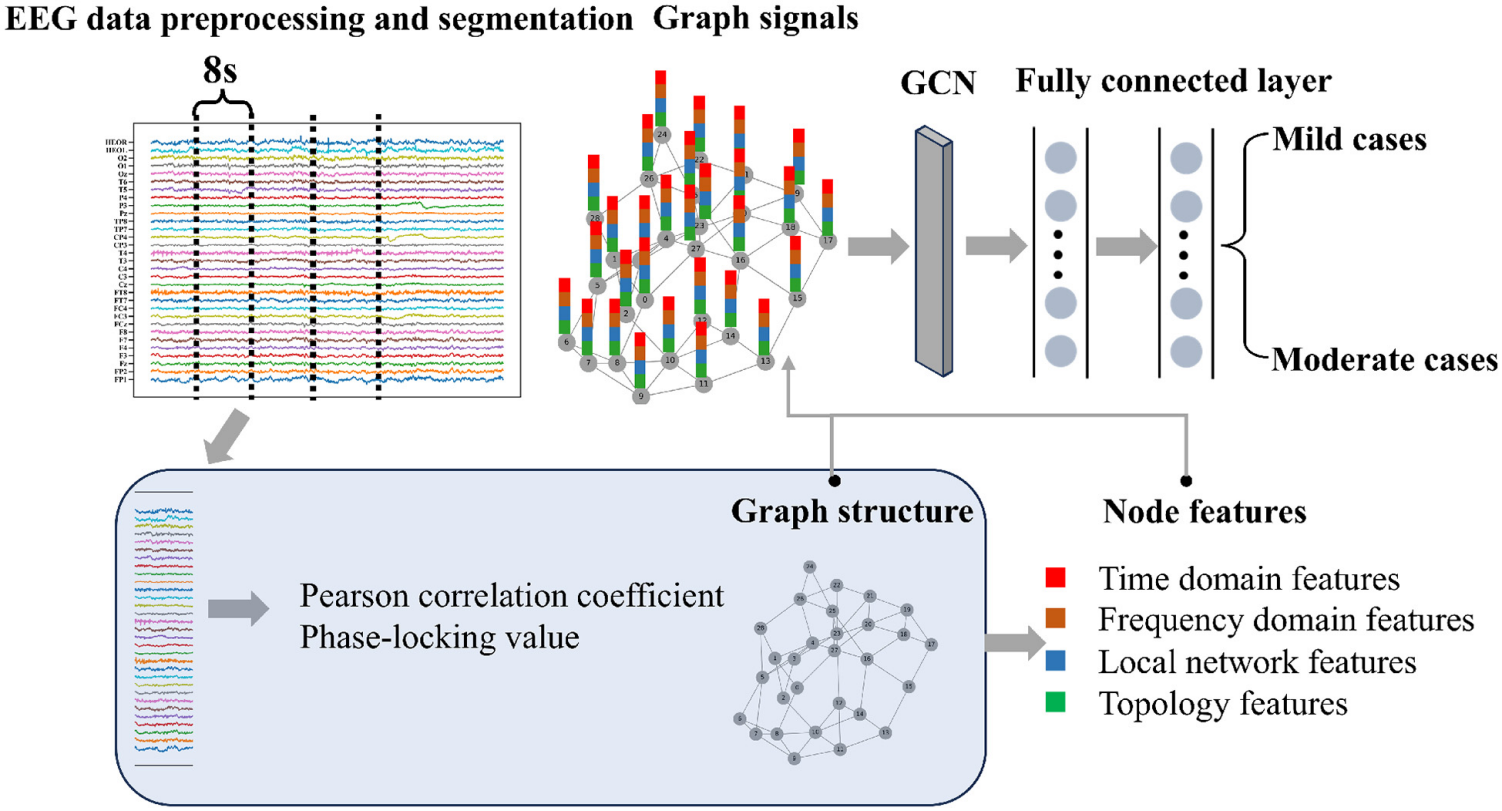
Pipeline of EEG data preprocessing and the graph convolutional network (GCN) framework. Graph signal construction: EEG signals are segmented and transformed into graph-structured data using connectivity measures (PCC, PLV). Node features: extracted features include time-domain, frequency-domain, local network, and topological features. GCN architecture: a single-layer GCN followed by two fully connected layers processes the graph signals for classification of mild and moderate cases.

In this study, a GCN was employed to identify mild and moderate patients, with the overall framework illustrated in Figure 2. The EEG signals of each patient were divided into five distinct frequency bands and segmented into continuous, non-overlapping 8-s windows. Each segment was a sample and subsequently used for feature extraction.

The key to constructing brain networks is selecting an appropriate functional connectivity metric. Commonly used measures include the Pearson correlation coefficient (PCC) (Friston, 1994) and phase-locking value (PLV) (Wang et al., 2019). This study tested each of these metrics to identify the one that maximizes classification accuracy.

The features initially considered for input into the GCN included time- and frequency-domain features, local brain network features, and higher-order features derived from topological data analysis (TDA). The time-domain features comprised the mean absolute value, standard deviation, skewness, and kurtosis (Al-Qazzaz et al., 2021; Mulyanto et al., 2019; Kusumastuti et al., 2021), while the frequency-domain feature was the power spectrum (Finnigan et al., 2007). Local brain network features included the clustering coefficient and betweenness centrality (Kim et al., 2020; Zhan et al., 2022). Additionally, higher-order features were extracted using persistent homology (PH), a key method in TDA that enables multiscale analysis of brain networks by identifying topological invariants.

**Figure 3:**
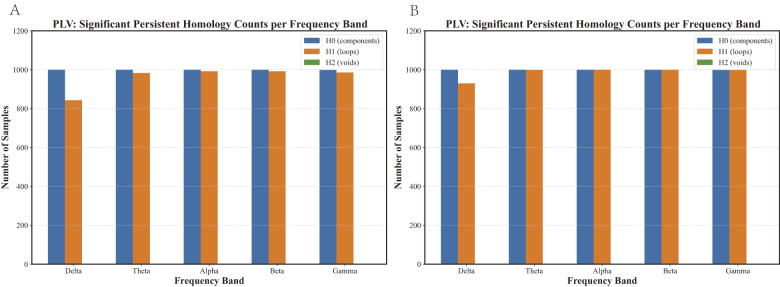
Number of samples with significant persistent homology features per frequency band. **(A)** Results for the PCC-based brain network. **(B)** Results for the PLV-based brain network. Only H0 (components) and H1 (cycles) were retained after denoising, whereas no significant H2 features were observed.

**Figure 4:**
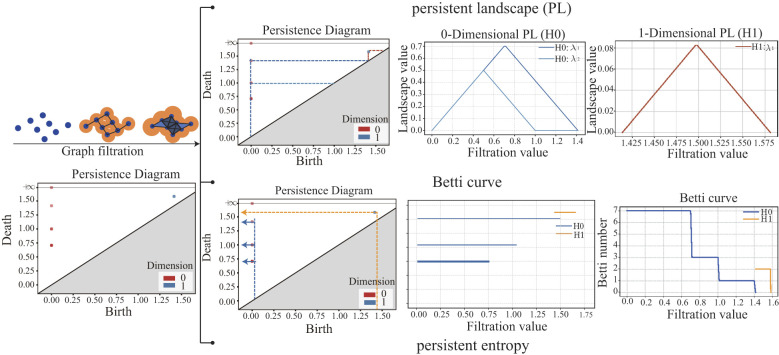
Topological data analysis (TDA) workflow for brain network characterization. Starting from graph filtration, persistent homology is computed to generate persistence diagrams. These diagrams are then quantitatively represented by persistent landscapes, Betti curves, and persistent entropy, enabling comprehensive and multi-perspective characterization of topological structures in the brain network.

The higher-order features were derived as follows: All samples exhibited only 0-dimensional (H0, connected components) and 1-dimensional (H1, cycles) topological features (Figure 3). Therefore, the higher-order features are constructed from the H0 and H1, with their dynamic evolution (birth–death processes of connected components/cycles) visualized and quantified via topological tools, as illustrated in Figure 4. First, distance matrices (Equation 1) were used to construct Vietoris–Rips (VR) complexes, which model how topological features emerge and vanish as a filtration parameter (e.g., edge length) increases. The resulting persistence diagrams plot each feature's birth (x-axis) against its death (y-axis), capturing its entire lifetime. These diagrams were then transformed into three quantifiable topological vectors. Persistent landscapes (Equation 2) (Bubenik, 2015): For each feature (H0/H1) with lifetime [*b*_*i*_, *d*_*i*_], a “triangular mountain” is defined: it rises linearly from birth *b*_*i*_ to the midpoint (*b*_*i*_+*d*_*i*_)/2, then falls back to 0 at death *d*_*i*_. At every filtration time *t*, the *k*_*th*_ landscape layer retains the *k*-tallest mountains (e.g., blue/red curves represent 0/1-dimensional landscapes as shown in Figure 4). This “multi-layered mountain range” quantifies how prominently features persist across growth stages. Betti curves (Equation 3) (Zomorodian and Carlsson, 2004): At each filtration time *t*, count how many features (H0/H1) are “alive” (i.e., *b*_*i*_ ≤ *t* ≤ *d*_*i*_). The resulting stepwise curves (e.g., blue/orange lines represent Betti curves as shown as in Figure 4) track how many topological features exist at every stage. Persistent entropy (Equation 4) (Atienza, 2019): This metric quantifies distributional complexity by normalizing each feature's lifetime (*d*_*i*_−*b*_*i*_) relative to the total persistence $\left(L_{D}=\underset{i}{\sum }\left(d_{i}-b_{i}\right)\right)$, yielding probabilities *p*_*i*_. High entropy implies diverse lifetimes, while low entropy suggests concentrated, dominant features. To further characterize global network properties, two amplitude metrics were computed: persistent landscape amplitude (integrating the area under all landscape layers, Equation 5) and Betti curve amplitude (integrating the area under Betti curves, Equation 6) (Garin and Tauzin, 2019). These, combined with persistent entropy, formed the final set of higher-order features.


$$\text{d}(a_{ij})=1-|a_{ij}]|,$$ (1)

where, d(*a*_*ij*_) is the element in distance matrix, and *a*_*ij*_ is the correlation value between brain nodes *i* and *j*.


$$\begin{array}{l} {\text{λ}}_{i}(t)=\{\begin{array}{ll} t-b_{i}, & \text{if }b_{i}\leq t\leq \frac{b_{i}+d_{i}}{2},\\ d_{i}-t, & \text{if }\frac{b_{i}+d_{i}}{2}<t\leq d_{i},\\ 0, & \text{otherwise,} \end{array}\\ {\text{λ}}_{k}(t)=\text{k-th largest value of }\{{\text{λ}}_{i}(t)\}. \end{array}$$ (2)

Here, (*b*_*i*_, *d*_*i*_) represents the birth time *b*_*i*_ and death time *d*_*i*_ of a topological feature.


$$\beta (t)=\#\{i|b_{i}\leq t\leq d_{i}\}.$$ (3)

Here, # means the number of homology classes that are alive at time *t*.


$$\begin{array}{l} H=-\sum_{i}p_{i}\log(p_{i}),\\ p_{i}=\frac{d_{i}-b_{i}}{L_{D}},\;L_{D}=\sum_{i}\left(d_{i}-b_{i}\right). \end{array}$$ (4)

$$\begin{array}{l} {\text{Amplitude}}_{\text{landscape}}=\|\text{λ}{\|}_{p}={\left(\sum_{k\in \mathbb{N}}{\left\|{\text{λ}}_{k}\right\|}_{p}^{p}\right)}^{1/p},\\ \;{\left\|{\text{λ}}_{k}\right\|}_{p}={\left(\sum_{j=1}^{N}{\text{λ}}_{k}{\left(t_{j}\right)}^{p}\right)}^{1/p}. \end{array}$$ (5)

Here, *p* represents the norm and *N* represents the number of layers. *t*_*j*_, *j* = 1, 2, …, *n* is a set of uniformly discretized sampling points covering the entire persistence graph (all births and deaths). We set *p* = 1 and *n* = 100, and calculated the amplitude of the persistent landscape for *N* = 1 and *N* = 2 respectively.


$${\text{Amplitude}}_{\text{Betti}}={\left\|\beta (t)\right\|}_{p}={\left(\sum_{j=1}^{n}\beta {\left(t_{j}\right)}^{p}\right)}^{1/p},$$ (6)

The meanings and values of *p* and *n* are the same as those defined in the persistent landscape amplitude. β(*t*_*j*_) is the Betti number at the discretized time point *t*_*j*_.

**Figure 5:**
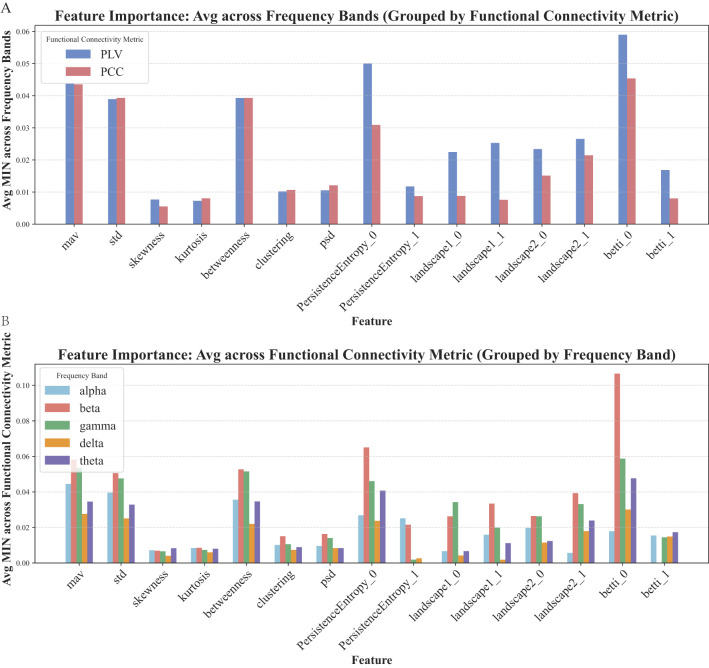
Feature importance based on MIN. **(A)** Average MIN scores of each feature across all frequency bands, grouped by functional connectivity metrics (PLV vs. PCC). **(B)** Average MIN scores across different functional connectivity metrics, grouped by frequency band (alpha, beta, gamma, delta, theta).

To assess the importance of each feature, mutual information (MIN) between each feature and the patients' labels was computed (Figure 5). The results indicated that the mean absolute value, standard deviation, power spectrum, betweenness centrality, and all higher-order features except PersistenceEntropy_1 contributed significantly to classification performance. The results were consistent across different functional connectivity metrics and EEG frequency bands. Consequently, these selected features were retained for final model training.

As a result of the feature selection, the final feature matrix for each sample had a shape of 29 (nodes) × 11 (features), comprising 2 time-domain features, 1 frequency-domain feature, 1 local network feature, and 7 higher-order features (excluding PersistenceEntropy_1). To address class imbalance—where the number of moderate stroke cases was half that of mild cases—the synthetic minority oversampling technique (SMOTE) was employed to oversample the minority class. Specifically, the SMOTE technique was implemented using the imbalanced-learn library (version 0.12.4) with the following settings: sampling_strategy='auto', k_neighbors=5 (default), and random_state=42. All features were normalized prior to being input into the GCN for classification.

We designed a lightweight graph convolutional neural network model, EEGGCN1, to perform EEG-based classification. The network architecture is summarized in Figure 2 and Table 2. The architecture consists of three key components: Graph Convolutional Block: A single-layer DenseGCNConv projects input features (*F* = 11) to a latent space of width *W* = 14, capturing channel-wise interactions through the adjacency matrix **A**∈ℝ^*B*×*N*×*N*^ (where *B* = 32, batch size). Batch normalization (BN) and LeakyReLU (α = 0.01) enhance training stability, followed by dropout to prevent overfitting. Specifically, the dropout rate *p* is set according to the frequency band and connectivity feature type. This adaptive dropout configuration aims to balance the regularization strength with the intrinsic variability of different frequency–feature combinations, thereby facilitating model convergence while avoiding both overfitting and underfitting. Feature Transformation: The GCN outputs (*B*×*N*×*W*) are flattened into node-embeddings of dimension *N*×*W* (406-D), preserving topological information while reducing dimensionality. Classification Block: Two fully connected (FC) layers further compress features: FC1 maps *N*×*W*→*N* (29-D) with LeakyReLU activation. FC2 reduces *N* to 2 for binary classification, finalized by log-softmax.

**Table 2:** EEGGCN1 architecture.

Block	Layer	# Units	Size	# Params	Output	Activation	Options
Input	Input	–	–	–	(*B, N, F*)	–	–
GCN	DenseGCNConv	*W*	(*F*→*W*)	*F*×*W*+*W*	(*B, N, W*)	Linear	–
	Reshape	–	(*B, N, W*) → (*B*×*N, W*)	–	(*B*×*N, W*)	–	–
	BatchNorm1d	–	–	2*W*	(*B*×*N, W*)	–	ϵ = 10^−5^
	LeakyReLU + Dropout	–	–	–	(*B*×*N, W*)	LeakyReLU	α = 0.01, *p*
	Reshape	–	(*B*×*N, W*) → (*B, N*×*W*)	–	(*B, N*×*W*)	–	–
FC	Linear	29	*N*×*W*→*N*	*N*×*W*×29+29	(*B, N*)	LeakyReLU	α = 0.01
	Dropout	–	–	–	(*B, N*)	–	*p*
	Linear	2	*N* → 2	*N*×2+2	(*B*, 2)	LogSoftmax	dim=1

*B* = batch size, *N* = 29 (nodes), *F* = 11 (input features), *W* = 14 (GCN width).

Optimal hyperparameters (learning rate, dropout) for each frequency band and connectivity measure:

**Table:** 

**Band**	**Connection**	**(lr, p)**
delta	PCC	(0.0050, 0.6)
	PLV	(0.0005, 0.6)
theta	PCC	(0.0005, 0.6)
	PLV	(0.0010, 0.5)
alpha	PCC	(0.0010, 0.5)
	PLV	(0.0050, 0.5)
beta	PCC	(0.0010, 0.4)
	PLV	(0.0050, 0.4)
gamma	PCC	(0.0050, 0.4)
	PLV	(0.0050, 0.4)

We trained the model using 10-fold cross-validation over 150 epochs. The fixed independent test set consisting of subjects with IDs 32, 48, 14, 20, 44, 60, 65, 7, 51, 28, 62, 10, 47, and 6 (subjects with IDs above 50 were generated via SMOTE) was used. To ensure reproducibility, all random processes (including data shuffling, weight initialization, and SMOTE resampling) were controlled using fixed random seeds. Specifically, we set the random seed to 42 for NumPy, PyTorch, and Scikit-learn operations. The initial learning rate was band-dependent. A step decay schedule was applied, reducing the learning rate by a factor of 10 every 50 epochs. Optimization was performed using the Adam optimizer, and the training objective was the cross-entropy loss. Training was performed on an Ubuntu 22.04 system with an NVIDIA RTX 2080 Ti GPU (11GB VRAM), using PyTorch 2.1.0 with CUDA 12.1 acceleration.

### External validation

To evaluate the generalizability of the proposed GCN classifier, external validation was performed on an independent cohort of 30 patients from Shanghai Pudong New Area Gongli Hospital, who met the same clinical inclusion criteria. This study, approved by the hospital's Institutional Review Board (GLYY1s2025-064), was conducted in accordance with the Declaration of Helsinki. All external EEG recordings were preprocessed using an identical pipeline to the primary dataset, encompassing filtering, segmentation, and feature extraction. The GCN model, trained solely on the primary dataset, was applied directly to this external cohort without any retraining or parameter adjustment. Performance was assessed using accuracy, precision, recall, F1-score, and AUC to ensure a fair comparison with the internal validation results.

### Additional experimental designs

#### Comparison with baseline classifiers

To comprehensively evaluate the effectiveness of the proposed EEGGCN1 framework, we further compared it with several representative baseline classifiers, including a random forest (RF), a two-layer convolutional neural network (CNN2) (Moon et al., 2020), EEGNet (Lawhern et al., 2018), regularized graph neural network (RGNN), and graph attention network (GAT). These baseline methods were implemented under the same experimental setting as EEGGCN1 to ensure a fair comparison. Specifically, all classifiers were trained and tested on the same dataset partition, and their performances were evaluated using the same metrics, including accuracy, F1-score, recall, and precision. For conventional and neural-network-based baselines, the input features were kept consistent with the corresponding experimental setting as much as possible. In particular, this comparison was intended to examine whether the graph-based modeling strategy adopted by EEGGCN1 could offer advantages over non-graph baselines, and to position its performance relative to representative graph-learning models.

#### Contribution of higher-order features

To quantify the contribution of higher-order topological information, an additional ablation study was conducted by constructing different feature configurations. Specifically, three feature settings were considered: local graph features only, higher-order topological features only, and the fusion of both feature groups. The local features mainly characterized node-level or pairwise connectivity properties derived from EEG functional networks, and time-frequency features, whereas the higher-order features captured global topological organization beyond pairwise interactions. These comparative experiments were designed to examine whether higher-order descriptors provided complementary discriminative information for stroke severity classification. All configurations were evaluated within the same classifier framework and under the same training-testing protocol, so that the observed differences could be attributed to the feature composition rather than changes in the model architecture or evaluation procedure.

#### Model explainability analysis

To improve the interpretability of EEGGCN1, SHAP (Shapley Additive Explanations) (Lundberg and Lee, 2017) was used to quantify the contribution of each input feature to the model predictions. Feature importance was ranked according to the mean absolute SHAP values.

#### Sensitivity analysis

To assess the robustness of the proposed model, we conducted sensitivity analyses on two key factors: sample length and EEG channel count.

Sample length variation. Each trial lasted 8 s, covering the instruction, MI, and break. To examine the influence of temporal windowing, we extracted contiguous segments of 2 s, 3 s, 4 s, 5 s, 6 s, 7 s, and 8 s, and evaluated the classification accuracy for each window using the same preprocessing and classification pipeline. Specifically: 2—4 s windows corresponded to the MI phase following cue onset. 5—6 s windows covered the MI phase and included part (1 s immediately preceding MI) or all of the instruction window. 7—8 s windows covered the entire instruction and motor imagery periods and included part (1 s immediately following MI) or all of the break window.

Channel count variation. To evaluate the effect of spatial sampling, we progressively increased the EEG channel number from 8 to 29, ensuring bilateral symmetry and approximate whole-brain coverage. The configurations were: 8-channel: Fp1, Fp2, C3, C4, O1, O2, Pz, Fz. 16-channel: + F3, F4, FC3, FC4, CP3, CP4, P3, P4. 22-channel: + F7, F8, T3, T4, T5, T6. 24-channel: + FCz, Oz. 29-channel: all available electrodes. All experiments used identical preprocessing, feature extraction, and model training protocols.

#### Alternative graph topology design

Because the graph topology directly determines information propagation in graph neural networks, we further evaluated the influence of alternative graph construction strategies on classification performance. In the current study, EEG functional connectivity matrices were first computed using PLV, and then different binarization strategies were adopted to obtain graph adjacency matrices with different sparsity levels. Specifically, four graph topology settings were considered: a fully connected graph and three threshold-based sparse graphs obtained by retaining PLV edges greater than or equal to 0.3, 0.5, and 0.8, respectively. The fully connected graph preserved all pairwise channel connections, whereas the thresholded graphs progressively removed weak functional connections and produced increasingly sparse topologies. Under each topology setting, the same EEGGCN1 architecture, training protocol, and evaluation metrics were used. This ablation was designed to assess whether the model performance depended on graph sparsity and to identify an appropriate graph construction strategy for the proposed framework.

### Cycle-based key brain regions

Cycle structures are considered to reflect higher-order information integration within brain networks (Chung et al., 2019). In this study, cycle-related node importance was quantified using the cycle ratio to characterize higher-order topological organization across different brain regions. Specifically, for each subject, frequency-specific and segment-wise EEG data were first used to compute distance matrices, from which Vietoris–Rips (VR) complexes were constructed with a maximum edge length threshold of 1.0 and a homology dimension up to 2. Persistent homology was then applied to each VR complex, and the homology class with the longest persistence (excluding classes persisting to infinity) was selected. The nodes and edges involved over the lifetime of this homology class were used to construct a corresponding adjacency matrix. Finally, the cycle ratio of each node was calculated following the method proposed by (Fan et al. 2021), which served as a node-level importance metric for subsequent cross-group comparison of brain region involvement.

## Results

### Classification performance under different frequency bands and functional connectivity metrics

The classification performance of EEGGCN1 across five frequency bands (delta, theta, alpha, beta, gamma) and two functional connectivity measures (PCC and PLV) is summarized in Table 3. Overall, the gamma band with PLV connectivity consistently yielded the best performance, achieving the highest values in accuracy (0.859), F1-score (0.871), recall (0.951), and precision (0.804). Across most bands, PLV outperformed PCC.

**Table 3:** Classification performance of EEGGCN1 across different frequency bands and connectivity measures (PCC vs. PLV).

Band	Method	Accuracy	F1-score	Recall	Precision
alpha	PCC	0.726 ± 0.017	0.764 ± 0.013	0.886 ± 0.015	0.671 ± 0.016
	PLV	0.763 ± 0.013	0.792 ± 0.009	0.905 ± 0.017	0.705 ± 0.016
beta	PCC	0.791 ± 0.011	0.818 ± 0.009	0.936 ± 0.013	0.726 ± 0.010
	PLV	0.806 ± 0.021	0.835 ± 0.014	**0.980 ± 0.006**	0.727 ± 0.023
delta	PCC	0.748 ± 0.015	0.774 ± 0.011	0.864 ± 0.011	0.702 ± 0.016
	PLV	0.675 ± 0.011	0.693 ± 0.012	0.734 ± 0.017	0.656 ± 0.010
gamma	PCC	0.809 ± 0.035	0.831 ± 0.026	0.933 ± 0.016	0.749 ± 0.040
	PLV	**0.859 ± 0.017**	**0.871 ± 0.013**	0.951 ± 0.006	**0.804 ± 0.024**
theta	PCC	0.651 ± 0.013	0.702 ± 0.009	0.822 ± 0.013	0.613 ± 0.012
	PLV	0.714 ± 0.013	0.756 ± 0.011	0.884 ± 0.018	0.660 ± 0.011

Bold values indicate the best performance for each metric.

**Figure 6:**
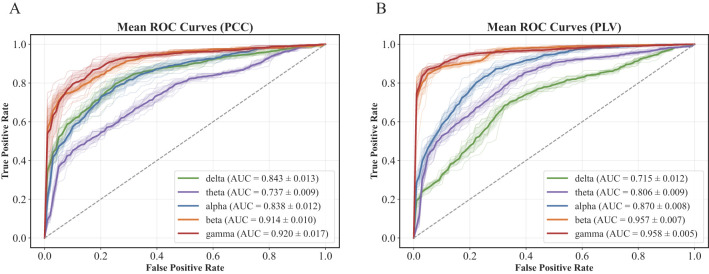
ROC curves for each frequency band under the two connectivity methods. **(A)** PCC and **(B)** PLV. The average AUC for each band is indicated in the legend.

To further support these findings, we visualized the Receiver Operating Characteristic (ROC) curves for all frequency-connectivity combinations. As shown in Figure 6, the gamma-PLV model exhibited the most favorable ROC profile, with the largest area under the curve, reflecting superior discrimination capability between mild and moderate stroke cases. Therefore, we selected the gamma-PLV setting as the default configuration for subsequent experimental analysis.

For completeness, the training and validation loss curves corresponding to all frequency band and connectivity metric combinations are presented in Supplementary Figures S1–S10. The training and validation loss curves were examined to identify parameter combinations that ensured stable convergence (the selected ranges highlighted by red boxes). Subsequently, the candidate hyperparameters were evaluated on the test set, and the configuration yielding the highest classification accuracy was chosen.

### External validation performance

**Figure 7:**
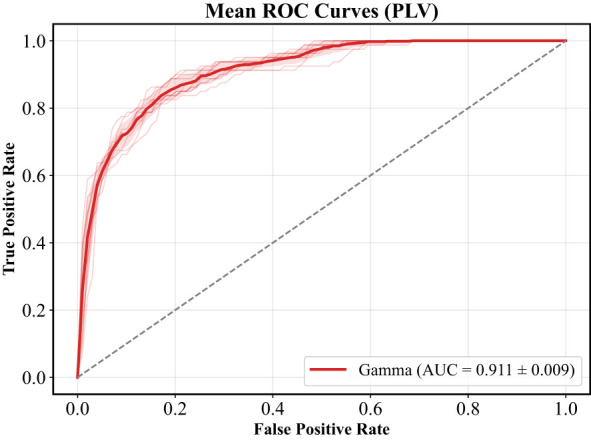
ROC curves of external validation.

On the independent external dataset, the proposed GCN classifier achieved values in accuracy (0.890), F1-score (0.622), recall (0.672), precision (0.590), and AUC (0.911, Figure 7). The comparable performance across datasets indicates that the proposed framework maintains stable discriminative capability when applied to EEG data collected at a different institution. Importantly, the evaluation on the external cohort was conducted using the same preprocessing pipeline and model parameters without retraining or fine-tuning, supporting the robustness and generalizability of the learned graph representations.

### Additional experimental results

#### Performance comparison of different classifiers

Compared with conventional machine learning models and other neural-network-based baselines, EEGGCN1 achieved superior or more balanced performance across multiple evaluation metrics, indicating the advantage of integrating EEG functional connectivity with graph convolutional learning. In particular, the graph-based formulation enabled the model to better exploit the topological organization of EEG channels, which is difficult to capture using conventional vector-based classifiers. The detailed quantitative results are summarized in Table 4.

**Table 4:** Performance comparison of different classifiers.

Methods	Accuracy	F1 Score	Recall	Precision
RF	0.753 ± 0.023	0.745 ± 0.025	0.753 ± 0.023	0.791 ± 0.022
CNN2	0.780 ± 0.028	0.814 ± 0.021	**0.961 ± 0.012**	0.706 ± 0.026
EEGNet	0.738 ± 0.070	0.738 ± 0.083	0.752 ± 0.111	0.736 ± 0.094
EEGGCN1	**0.859 ± 0.017**	**0.871 ± 0.013**	0.951 ± 0.006	**0.804 ± 0.024**
RGNN	0.844 ± 0.031	0.861 ± 0.025	0.961 ± 0.023	0.781 ± 0.038
GAT	0.826 ± 0.023	0.843 ± 0.019	0.929 ± 0.015	0.771 ± 0.025

Bold values indicate the best performance for each metric.

#### Contribution of higher-order features to classification

The results demonstrated that higher-order topological features provided useful discriminative information for stroke severity classification. Although local and higher-order features each showed predictive value, their combination yielded the best overall performance, suggesting a complementary effect between the two feature types. As detailed in Table 5.

**Table 5:** Performance comparison under local and higher-order feature matrices.

Feature matrix	Accuracy	F1 Score	Recall	Precision
Local only	0.783 ± 0.016	0.810 ± 0.012	0.929 ± 0.014	0.719 ± 0.019
Higher-order only	0.833 ± 0.020	0.847 ± 0.017	0.926 ± 0.018	0.782 ± 0.022
Local + Higher-order fusion	**0.859 ± 0.017**	**0.871 ± 0.013**	**0.951 ± 0.006**	**0.804 ± 0.024**

Bold values indicate the best performance for each metric.

#### Explainability analysis

**Figure 8:**
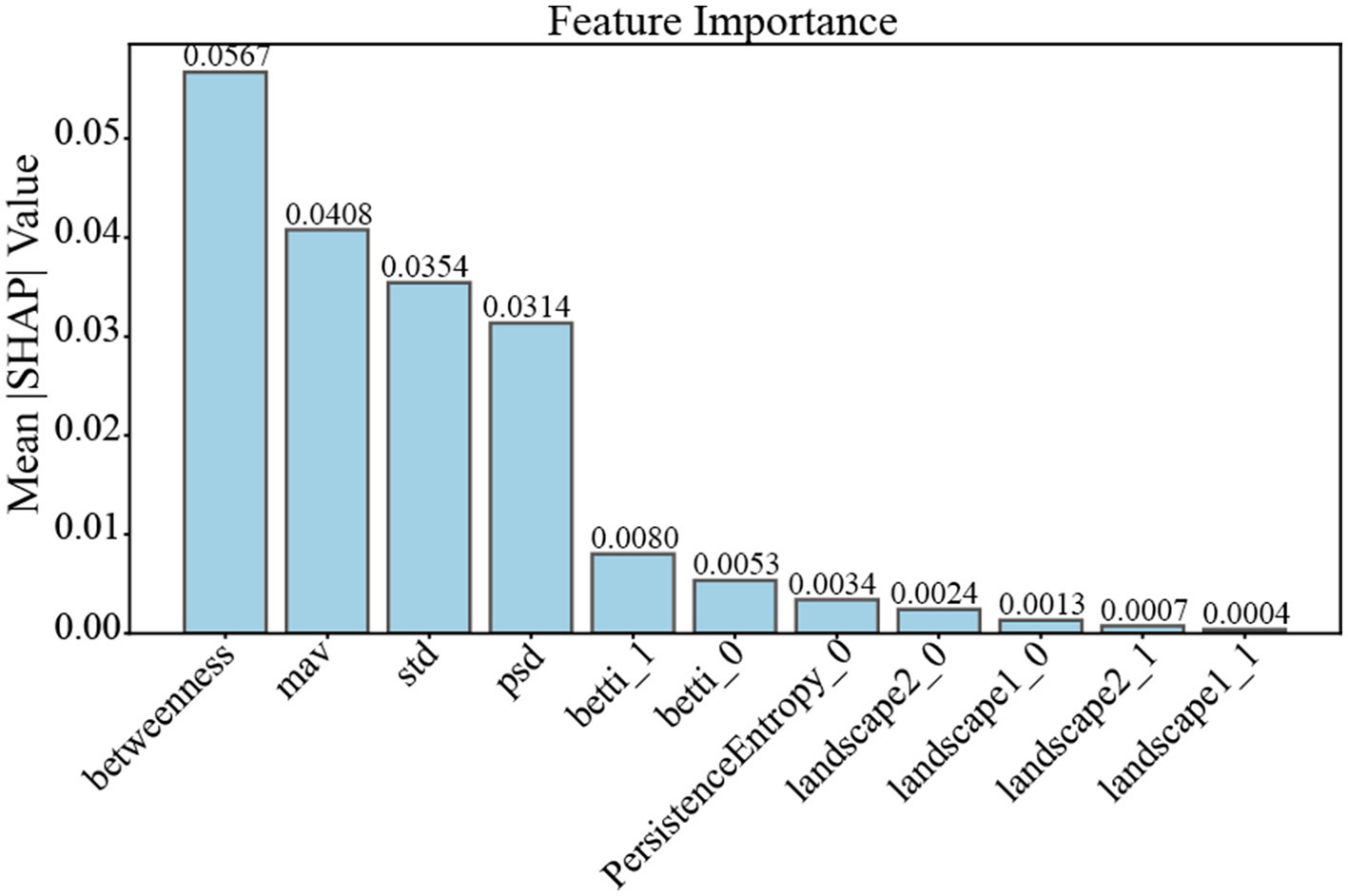
SHAP-based feature importance analysis of gamma PLV in stroke motor imagery decoding.

As shown in Figure 8, conventional EEG features, particularly betweenness centrality, maximum value, standard deviation, and spectral power density, exhibit relatively large SHAP values. This indicates that these features play a prominent role in driving the final classification decision once graph representations have been learned. In contrast, higher-order topological descriptors derived from persistent homology, including Betti numbers, persistence entropy, and persistence landscapes, display smaller SHAP values.

Importantly, the lower SHAP magnitudes of higher-order features do not imply limited relevance. Rather, they suggest that these features contribute to the model in a more indirect manner by constraining and stabilizing the learned graph representations. Consistent with this interpretation, classification experiments using only higher-order features achieved higher accuracy than those using only conventional features, highlighting the complementary roles of local and higher-order information.

Overall, the SHAP analysis reveals a multi-scale decision mechanism of the proposed GCN framework: local network and spectral features predominantly drive the decision boundary, while higher-order topological features provide global structural context that enhances the robustness and discriminative power of the learned representations.

#### Sensitivity analysis results

**Figure 9:**
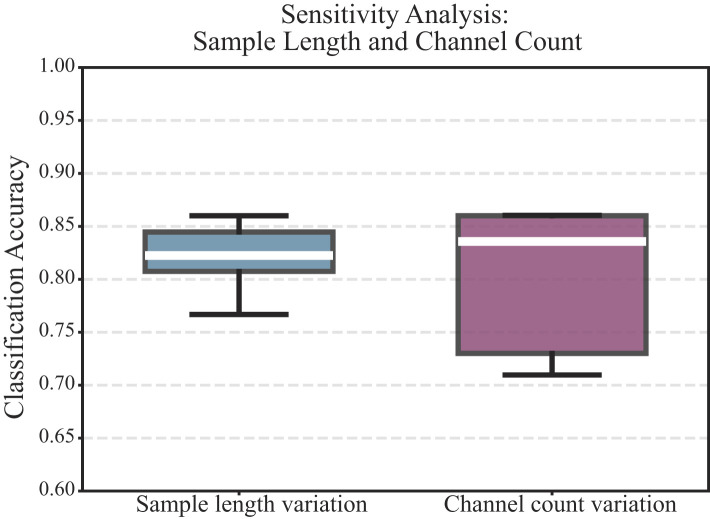
Sensitivity analysis of classification accuracy with respect to sample length and channel count. **(Left)** accuracy for time windows of 2—8 s. **(Right)** accuracy for channel configurations of 8, 16, 22, 24, and 29 channels. Boxplots reflect cross-validation variability.

The sensitivity analysis results are summarized in Figure 9. Sample length variation (2—8 s): Classification accuracies were [0.77, 0.80, 0.82, 0.8225, 0.84, 0.85, 0.86]. Accuracy increased monotonically with longer sample length, from 0.77 (2 s) to 0.86 (8 s). Performance improved markedly between 2—4 s and reached a plateau after 6 s, indicating that including both the instruction and MI windows substantially improves discriminative information, while adding partial break yields marginal gains. Channel count variation (8—29 channels): Accuracies for 8, 16, 22, 24, and 29 channels were [0.71, 0.73, 0.84, 0.86, 0.86]. Accuracy rose modestly from 8 to 16 channels, then sharply improved at 22 channels and saturated thereafter.

#### Ablation results on alternative graph topologies

The ablation study on alternative graph topologies showed that the graph construction strategy had a clear impact on classification performance (Table 6). In general, moderately sparse PLV-based graphs achieved better overall results than either the fully connected graph or overly sparse graphs, indicating that appropriate sparsification can improve the discriminability of EEG functional networks. Although some stricter thresholds yielded competitive performance on certain metrics, the topology constructed with a PLV threshold of 0.3 showed smaller variance across repeated runs and more stable overall performance. Therefore, the threshold of 0.3 was retained as the default graph construction setting in this study.

**Table 6:** Ablation results of EEGGCN1 under alternative PLV-based graph topology construction strategies.

Graph topology	Accuracy	F1-score	Recall	Precision
Fully connected	0.711 ± 0.031	0.734 ± 0.082	0.838 ± 0.170	0.681 ± 0.063
PLV ≥0.3	0.859 ± 0.017	0.871 ± 0.013	0.951 ± 0.006	0.804 ± 0.024
PLV ≥0.5	**0.874 ± 0.025**	**0.887 ± 0.020**	0.980 ± 0.006	**0.811 ± 0.034**
PLV ≥0.8	0.791 ± 0.015	0.826 ± 0.010	**0.992 ± 0.002**	0.708 ± 0.015

Bold values indicate the best performance for each metric.

#### Key brain regions

**Figure 10:**
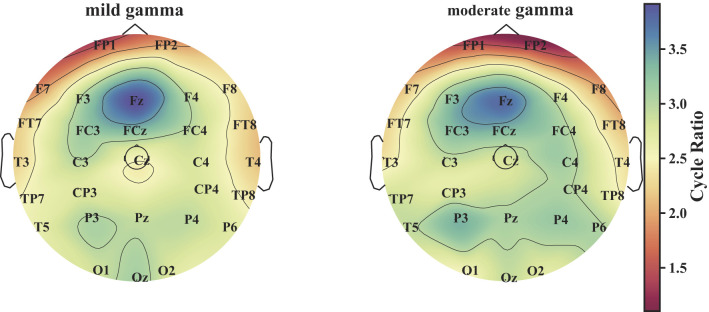
The cycle ratio-weighted brain maps of patients with mild and moderate IS.

Figure 10 shows the spatial distribution of node-level cycle ratios derived from persistent homology. Prefrontal regions consistently exhibited the highest cycle ratios among all areas. Statistical comparison revealed no significant differences in regional cycle ratios between mild and moderate stroke patients (*p* > 0.05). These results indicate that prefrontal regions are prominently involved in cycle-related higher-order network structures.

## Discussion

This study makes several key contributions to EEG-based stroke severity classification. First, we introduce a higher-order topological perspective for brain network analysis by leveraging persistent homology, which enables the characterization of stable cycle structures beyond conventional pairwise connectivity. This framework alleviates the dependence on manually selected thresholds and captures global network organization associated with recurrent information pathways. Second, by embedding a comprehensive feature representation into a graph convolutional network, the proposed EEGGCN1 framework effectively combines topological data analysis with graph-based learning, yielding robust classification performance across frequency bands and connectivity measures. The best performance is achieved under the gamma band with PLV-based connectivity, reaching an accuracy of 86% in distinguishing mild and moderate stroke patients. Finally, cycle-based analysis identifies the prefrontal cortex as a key region involved in higher-order network organization, highlighting the potential of topological features for revealing clinically relevant brain regions beyond traditional spectral and connectivity measures.

The gamma band is widely recognized for its role in high-level cognitive processes, including attention, perception, and working memory. The better classification performance observed in this band suggests that disruptions in high-frequency cortical dynamics are particularly sensitive to stroke-related neural impairments. In the context of motor imagery tasks, the dorsolateral prefrontal cortex has been consistently identified as a core node within the motor-cognitive network (Dux et al., 2009). This network-centric view is supported by extensive neuroimaging and neuromodulation studies (Vry et al., 2012; Wu et al., 2018; Martel and Glover, 2023), underscoring the role of prefrontal activity in modulating motor representations. From a network-topological perspective, our cycle-based analysis further complements these findings by showing that prefrontal regions consistently exhibit high involvement in cycle-related higher-order structures. Although no statistically significant group-wise differences in regional cycle ratios were detected, the prominent participation of frontal nodes (e.g., Fz, F3, and F4) in cycle structures suggests that stroke-related alterations may affect the higher-order organization of prefrontal functional networks. This observation provides a potential link between spectral-domain abnormalities and network-level structural organization. Together, these results suggest that gamma-band network topology in prefrontal regions represents a promising dimension for characterizing functional alterations after stroke, offering complementary information beyond conventional spectral and connectivity measures.

The superior performance of EEGGCN1 compared to traditional classifiers such as RF, CNN2, and EEGNet underscores the importance of domain-specific feature engineering and topological representation learning. EEGNet, which directly processes raw EEG time series, performed poorly, likely due to its sensitivity to noise and lack of physiologically meaningful constraints. In contrast, models using carefully extracted features—such as PLV connectivity and spectral metrics—showed greater robustness and better generalization. Beyond graph-based approaches, the application of Transformer models to EEG signals presents a compelling alternative for capturing complex spatio-temporal dynamics. A particularly relevant implementation for our context would involve treating each electrode's time-series as a token, using the spatial coordinates of the EEG as 3D positional encodings. This formulation would allow the self-attention mechanism to directly model non-linear interactions between distant brain regions—something that might complement our persistence homology features that capture higher-order cyclical structures. However, several domain-specific challenges would need addressing: First, the quadratic complexity of attention is particularly problematic for long EEG sequences; this might require specialized attention mechanisms (e.g., Performer, Linformer) or segment-based processing. Second, unlike natural language, EEG tokens lack semantic discreteness, making the learned attention weights more difficult to interpret neurophysiologically. A promising research direction would be to integrate the topological priors we identified—such as the importance of prefrontal cycle structures in the gamma-band—directly into the Transformer architecture, perhaps through a hybrid model that uses graph-based embeddings as initial token representations or that incorporates persistent homology as a regularization term to guide the attention mechanism toward biologically plausible connectivity patterns. This could bridge the gap between data-driven attention maps and our topology-driven findings, potentially leading to more interpretable and physiologically grounded models for stroke severity assessment.

Beyond classification performance, the ability to distinguish between mild and moderate stroke has direct clinical relevance. In acute settings, rapid and objective assessment of stroke severity is critical for treatment decisions—such as eligibility for thrombolysis or mechanical thrombectomy—and for determining rehabilitation intensity. Our model, based on non-invasive EEG and topological features, could serve as a complementary tool to the clinical NIHSS assessment, particularly when experienced neurologists are unavailable or when serial monitoring is needed. It provides a quantitative, data-driven layer of information that may reduce inter-rater variability and support more consistent patient stratification.

Compared with prior work that adopted a four-class severity classification (e.g., (Nurfirdausi et al., 2022), reporting 83% accuracy across four NIHSS-based categories), our binary approach offers both advantages and trade-offs. Finer-grained classification can capture a more nuanced spectrum of impairment, which may be valuable for personalized rehabilitation planning. However, binary stratification aligns with key clinical decision thresholds (mild: NIHSS ≤ 4, often managed conservatively; moderate: NIHSS 5—15, typically candidates for intensive intervention and monitoring) and reduces class imbalance issues that complicate multi-class learning. It is also worth noting that our binary classification encompasses the vast majority of acute ischemic stroke patients. Epidemiological studies indicate that approximately 70–80% of stroke patients present with mild to moderate deficits (NIHSS ≤ 15), while severe strokes (NIHSS ≥ 15) are less common and often clinically obvious due to profound neurological impairments (P A Lyrer, 2009).

The proposed EEGGCN1 framework is intended to complement, rather than replace, established clinical severity assessment (e.g., NIHSS) by providing an objective EEG-derived marker of functional network integrity. EEG is portable and cost-effective, which may facilitate bedside monitoring and rehabilitation stratification when repeated neuroimaging is impractical. Several results support translational feasibility. First, we performed external validation on an independent hospital cohort by directly applying the model trained on the primary dataset without retraining or parameter tuning, demonstrating stable discrimination across institutions. Second, sensitivity analyses indicate practical scalability under constrained acquisition: performance increased with longer segments and with additional channels but saturated beyond a moderate montage size, suggesting that high-density EEG may not be strictly required for deployment. Nonetheless, broader clinical adoption will require larger multi-center prospective studies with heterogeneous devices and protocols, standardized artifact/quality-control procedures.

Despite the promising results, several limitations should be acknowledged. First, the sample size in this study is relatively modest, particularly for the moderate stroke group (*n* = 17) compared to the mild group (*n* = 33). Although SMOTE was applied to address class imbalance during training, a larger and more balanced cohort would enhance the statistical power and generalizability of the findings. Second, we adopted a binary classification scheme (mild vs. moderate) based on NIHSS scores. While this stratification aligns with common clinical decision points, it does not capture the full spectrum of stroke severity, including severe cases (NIHSS > 15). Future studies should explore finer-grained classification or regression approaches to predict continuous severity scores. Third, the current analysis is cross-sectional and lacks longitudinal follow-up data. Consequently, the model's ability to predict functional recovery or monitor disease progression over time remains unknown. Longitudinal studies are needed to assess whether the proposed topological features can serve as prognostic biomarkers.

## Data Availability

The original contributions presented in the study are included in the article/Supplementary material, further inquiries can be directed to the corresponding author.
