# Supplementary material for: EEG-based stroke severity classification using higher-order topological features and graph convolutional networks
Source: Front Neurosci. 2026 Apr 17;20:1791960. doi: 10.3389/fnins.2026.1791960 (PMC13133680; doi:10.3389/fnins.2026.1791960)
Supplement: Supplementary file 1 [file Data_Sheet_1.pdf]

Supplementary Material

The training and validation loss curves corresponding to all frequency band and connectivity metric combinations are presented in Appendix Figures S1-S10. The training and validation loss curves were examined to identify parameter combinations that ensured stable convergence (the selected ranges highlighted by red boxes). Subsequently, the candidate hyperparameters were evaluated on the test set, and the configuration yielding the highest classification accuracy was chosen (Tables S1-S5).

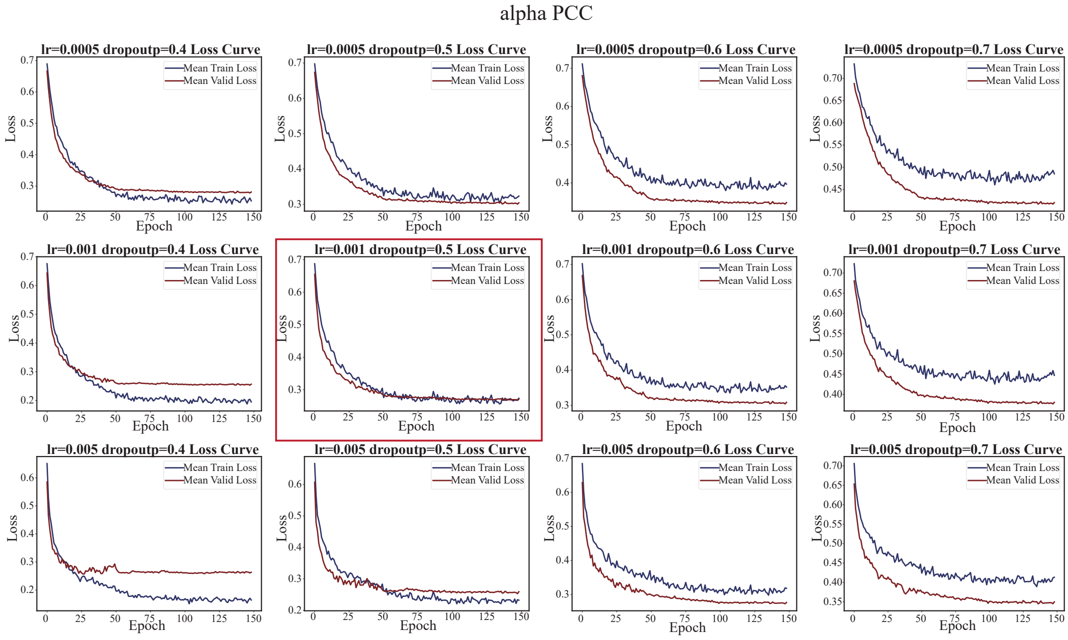

Figure S1. Loss curve for the alpha band using PCC connectivity metrics.

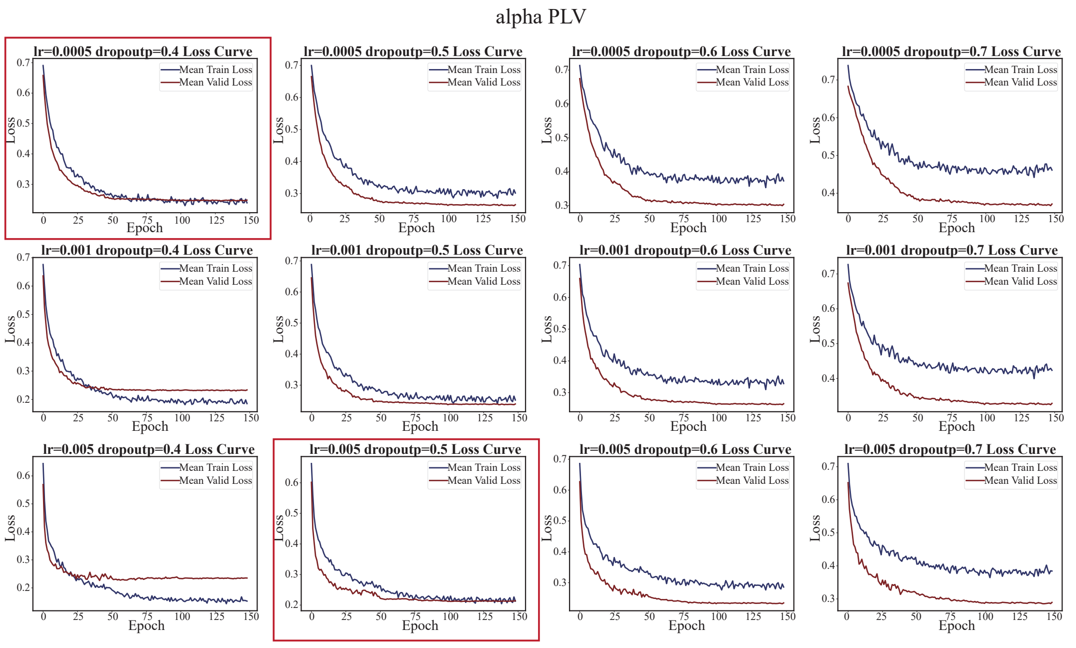

Figure S2. Loss curve for the alpha band using PLV connectivity metrics.

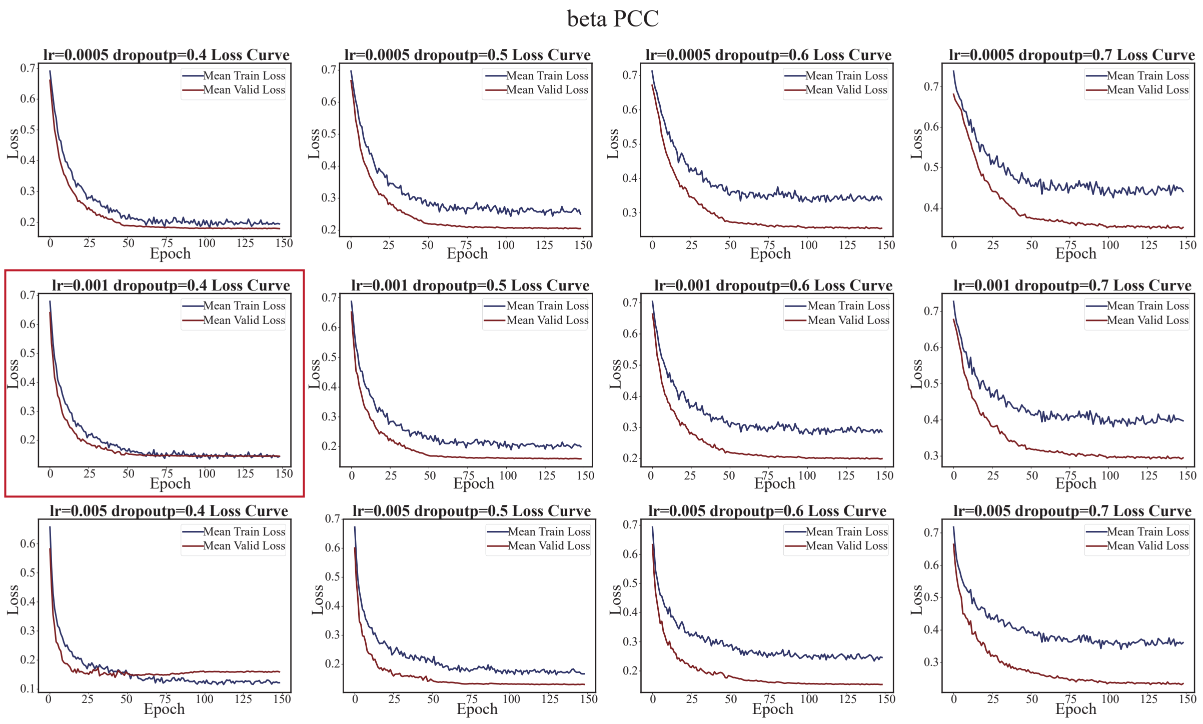

Figure S3. Loss curve for the beta band using PCC connectivity metrics.

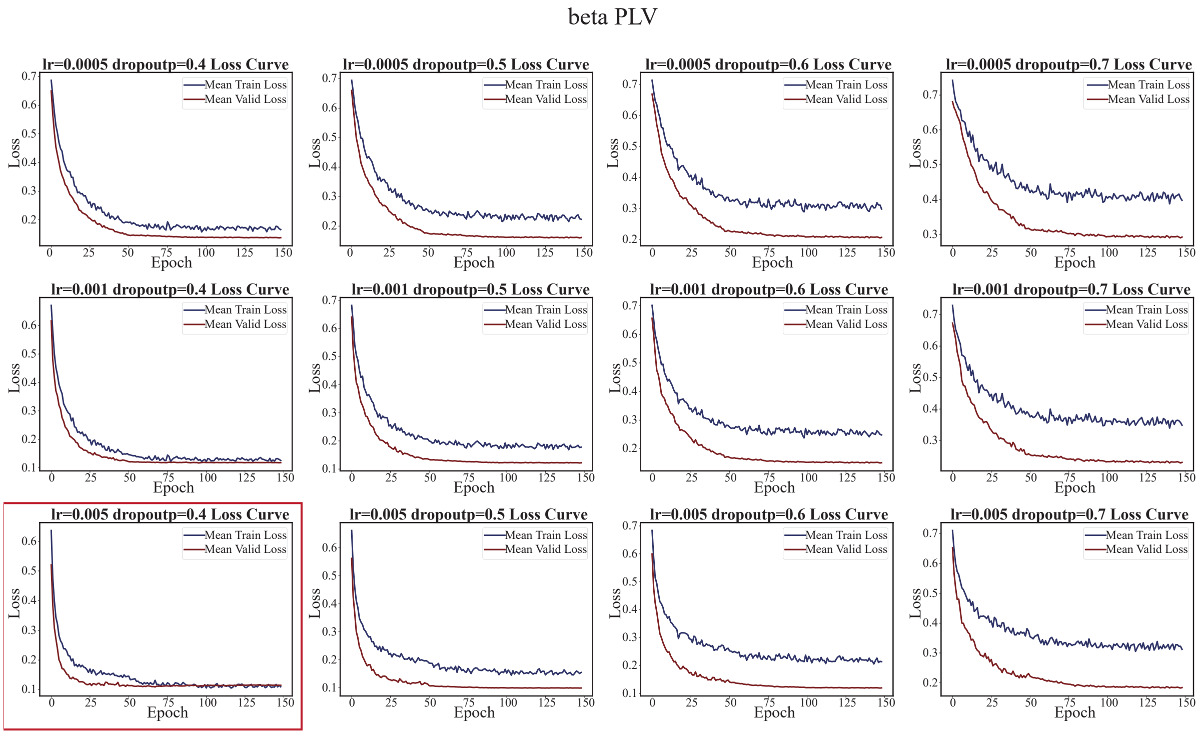

Figure S4. Loss curve for the beta band using PLV connectivity metrics.

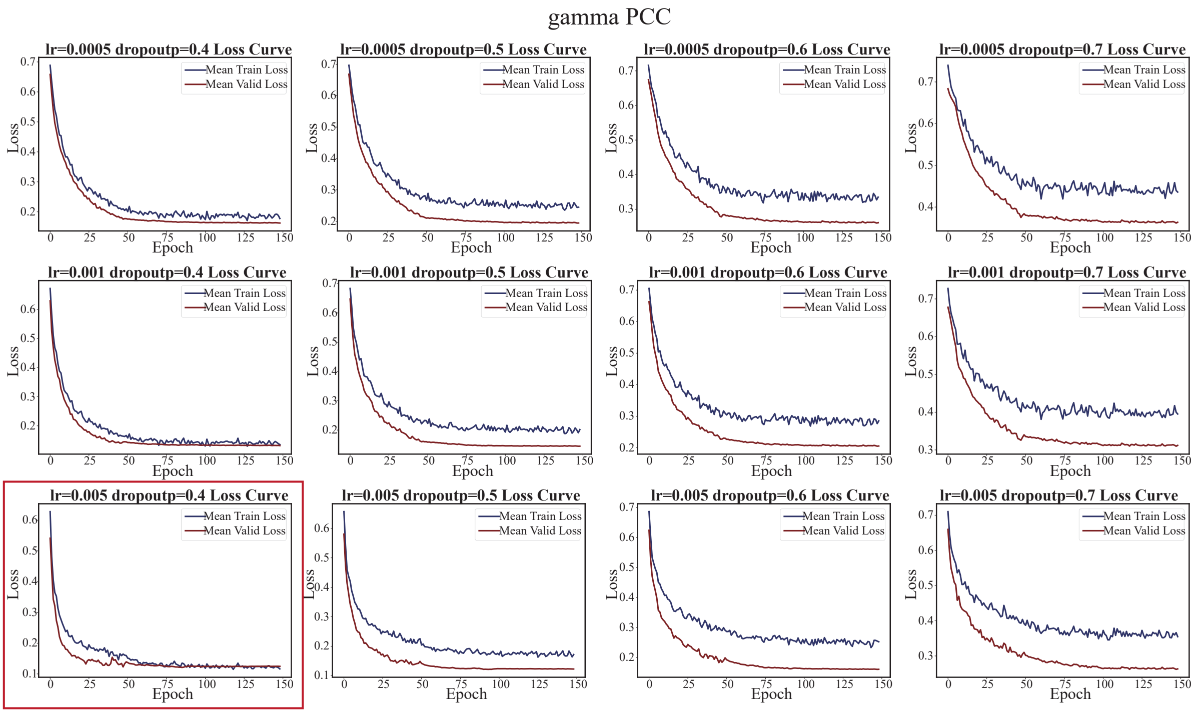

Figure S5. Loss curve for the gamma band using PCC connectivity metrics.

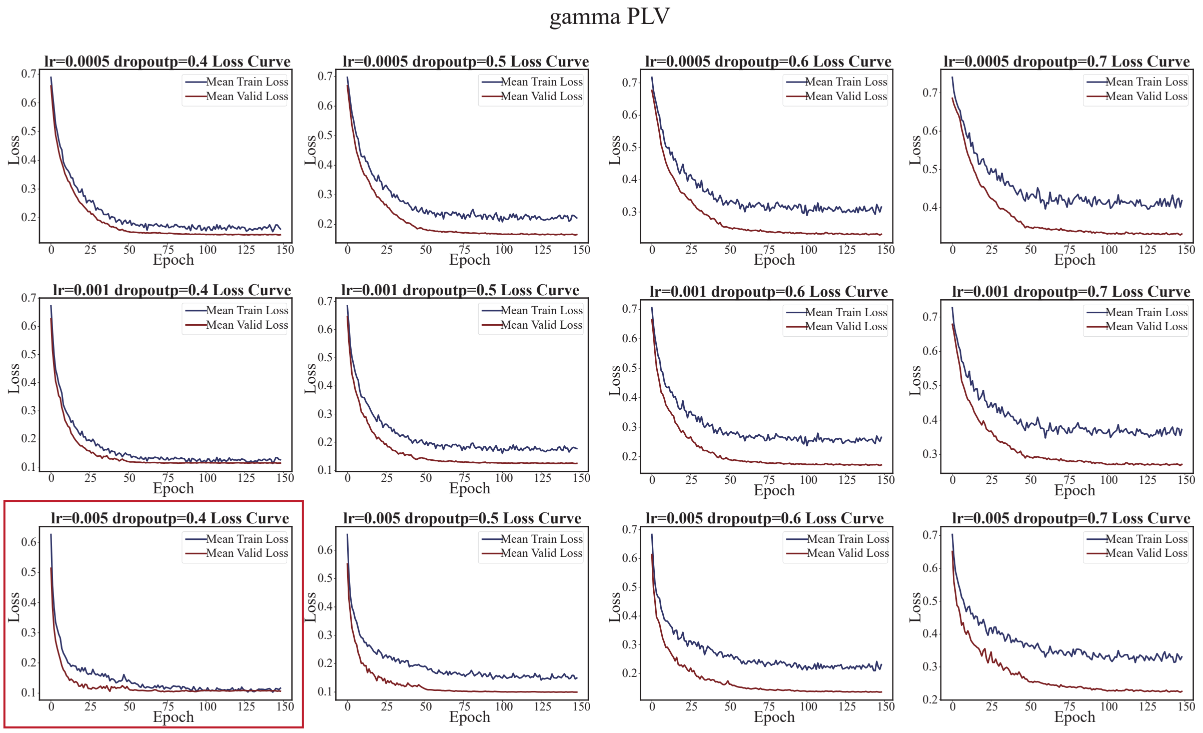

Figure S6. Loss curve for the gamma band using PLV connectivity metrics.

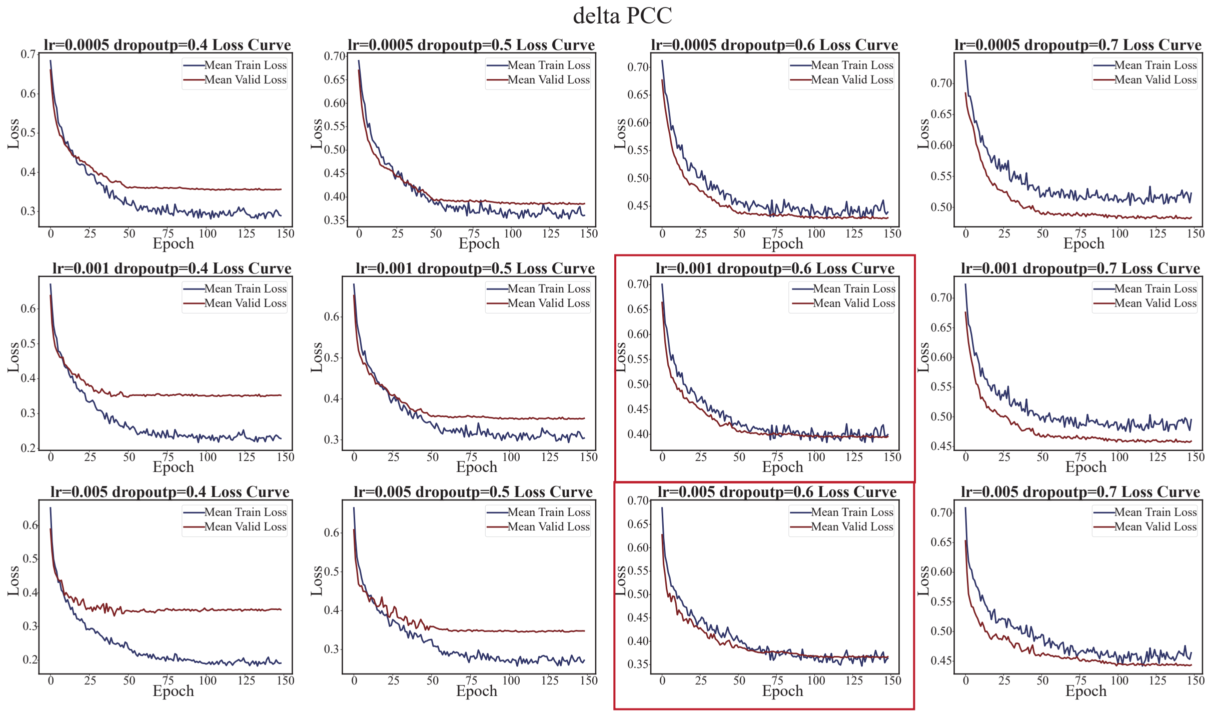

Figure S7. Loss curve for the Delta band using PCC connectivity metrics.

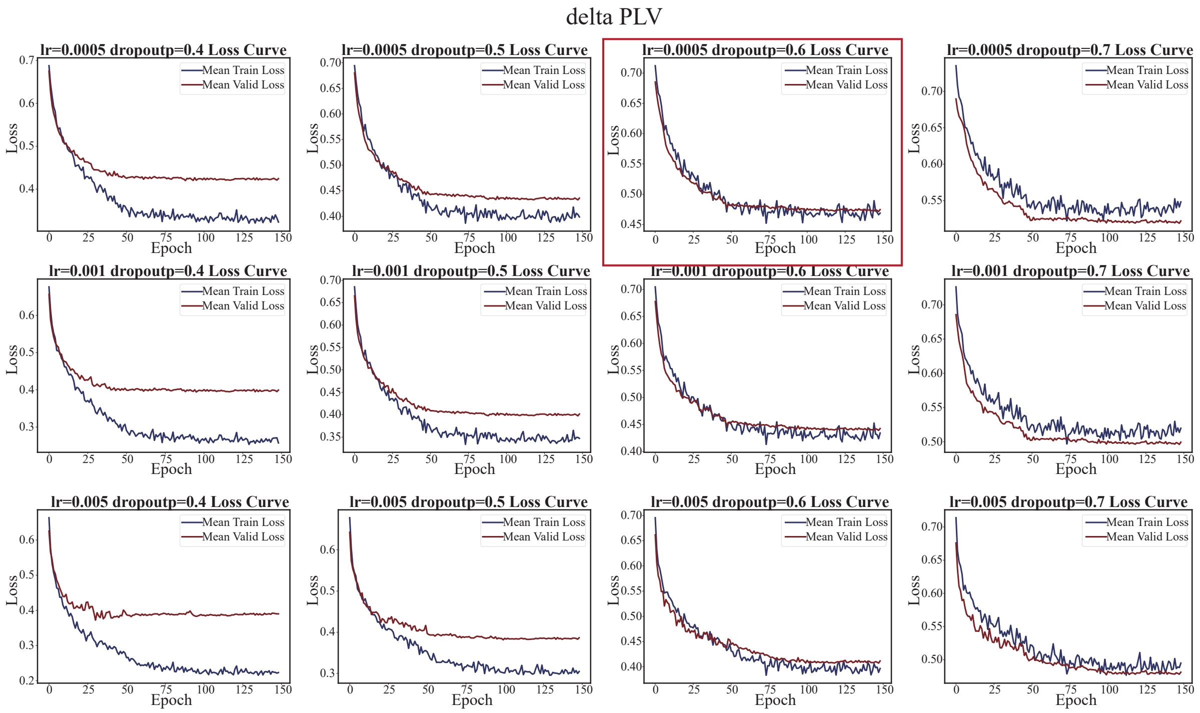

Figure S8. Loss curve for the Delta band using PLV connectivity metrics.

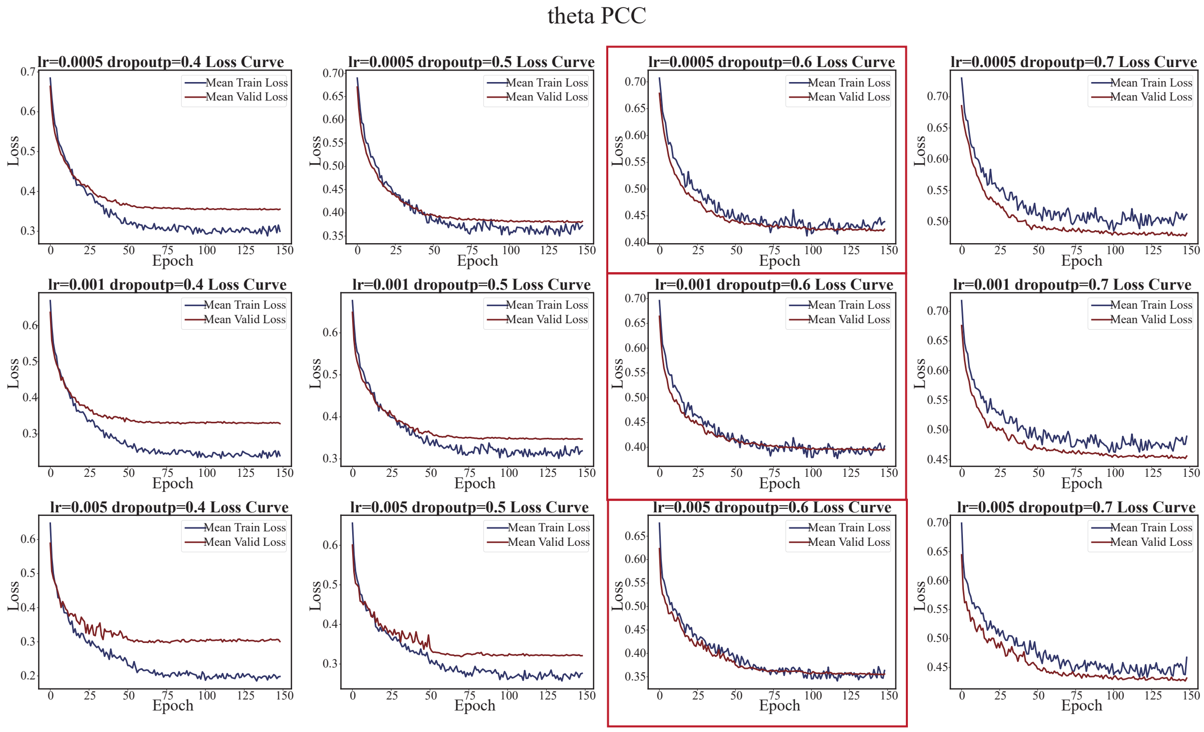

Figure S9. Loss curve for the theta band using PCC connectivity metrics.

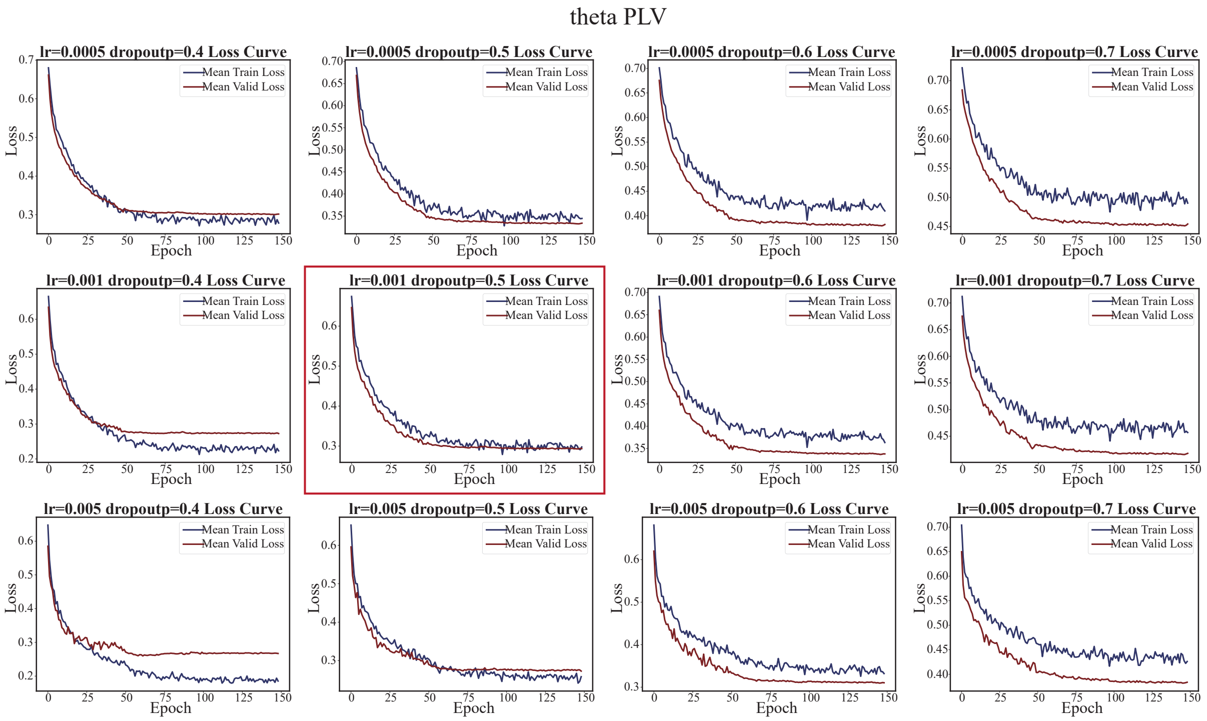

Figure S10. Loss curve for the theta band using PLV connectivity metrics.

Table S1. Model performance under different hyperparameters for the alpha band.

Band	Connection	Learning rate	Dropoutp	Accuracy	F1-score	Recall	Precision
alpha	PCC	0.0010	0.5	0.726 ± 0.017	0.764 ± 0.013	0.886 ± 0.015	0.671 ± 0.016
	PLV	0.0005	0.4	0.754 ± 0.014	0.780 ± 0.012	0.872 ± 0.016	0.705 ± 0.013
		0.0050	0.5	**0.763** ± 0.013	0.792 ± 0.009	0.905 ± 0.017	0.705 ± 0.016

Table S2. Model performance under different hyperparameters for the beta band.

Band	Connection	Learning rate	Dropoutp	Accuracy	F1-score	Recall	Precision
beta	PCC	0.0010	0.4	0.791 ± 0.011	0.818 ± 0.009	0.936 ± 0.013	0.726 ± 0.010
	PLV	0.0050	0.4	**0.806** ± 0.021	0.835 ± 0.014	0.980 ± 0.006	0.727 ± 0.023

Table S3. Model performance under different hyperparameters for the gamma band.

Band	Connection	Learning rate	Dropoutp	Accuracy	F1-score	Recall	Precision
gamma	PCC	0.0050	0.4	0.809 ± 0.035	0.831 ± 0.026	0.933 ± 0.016	0.750 ± 0.040
	PLV	0.0050	0.4	**0.859** ± 0.017	0.871 ± 0.013	0.951 ± 0.006	0.804 ± 0.024

Table S4. Model performance under different hyperparameters for the Delta band.

Band	Connection	Learning rate	Dropoutp	Accuracy	F1-score	Recall	Precision
Delta	PCC	0.0010	0.6	0.738 ± 0.013	0.762 ± 0.011	0.842 ± 0.015	0.696 ± 0.011
		0.0050	0.6	**0.748** ± 0.015	0.774 ± 0.011	0.864 ± 0.011	0.702 ± 0.016
	PLV	0.0005	0.6	0.675 ± 0.011	0.693 ± 0.012	0.734 ± 0.017	0.656 ± 0.010

Table S5. Model performance under different hyperparameters for the theta band.

Band	Connection	Learning rate	Dropoutp	Accuracy	F1-score	Recall	Precision
theta	PCC	0.0005	0.6	0.651 ± 0.013	0.702 ± 0.009	0.822 ± 0.013	0.613 ± 0.012
		0.0010	0.6	0.649 ± 0.019	0.706 ± 0.012	0.843 ± 0.017	0.608 ± 0.016
		0.0050	0.6	0.648 ± 0.028	0.712 ± 0.018	0.871 ± 0.018	0.603 ± 0.022
	PLV	0.0010	0.5	**0.714** ± 0.013	0.756 ± 0.011	0.884 ± 0.018	0.660 ± 0.011
